# The Effect of Upward Educational Mobility on Cognitive Performance Among Older Adults: A Comparison Between Mexico and South Africa

**DOI:** 10.1002/alz.087550

**Published:** 2025-01-09

**Authors:** Erika Meza

**Affiliations:** ^1^ Harvard University, Cambridge, MA USA

## Abstract

**Background:**

Upward socioeconomic trajectories across the life course have been protectively associated with late‐life cognitive outcomes. However, few studies have explored this association in low‐ and middle‐income countries (LMICs). Our study examines how upward educational mobility affects cognitive performance in nationally representative samples of older adults in Mexico and South Africa, two LMICs with a history of social and economic disadvantages and different educational transformations.

**Method:**

Data were from two nationally representative samples of adults 50 years and older, the Mexican Health and Aging Study (MHAS), the Health and Aging in Africa Longitudinal Study in South Africa (HAALSI), and their harmonized cognitive aging ancillary studies, Mex‐Cog and HAALSI‐Dementia. We used linear regression models to evaluate the relationship between upward educational mobility in adjacent generations (i.e., first‐generation to receive a formal education) and cognitive performance (i.e., orientation, executive function, language, memory, and global cognitive domains).

**Result:**

Our study included 1,031 participants whose parents did not receive any formal education; most were female (58%), and on average Mex‐Cog participants (n = 584) were slightly younger (mean: 69; sd: 9.3) than HAALSI‐dementia participants (n = 447; mean: 72; sd: 11.3). Among individuals whose parents did not receive a formal education, participants who received some education (i.e., upwardly mobile) had higher cognitive performance scores at baseline. Adjusting for age and sex, upwardly mobile individuals had 0.52 (95% CI: 0.42, 0.63) higher orientation, 0.39 (95% CI: 0.29, 0.48) higher language, and 0.37 (95% CI: 0.29, 0.46) higher memory performance in both cohorts. Greater benefits of upward educational mobility on executive function and global cognitive performance were observed for Mex‐Cog participants (B: 0.79; 95% CI: 0.67, 0.92 and B: 0.66; 95% CI: 0.54, 0.78, respectively) compared to their HAALSI‐dementia counterparts (B: 0.44; 95% CI: 0.33, 0.56 and B: 0.51; 95% CI: 0.39, 0.62).

**Conclusion:**

Our findings emphasize the impact of greater access to educational opportunities on late‐life cognitive performance and the importance of contextualizing educational transformational benefits in different settings.

